# A Shoe-Embedded Piezoelectric Energy Harvester for Wearable Sensors

**DOI:** 10.3390/s140712497

**Published:** 2014-07-11

**Authors:** Jingjing Zhao, Zheng You

**Affiliations:** 1 Collaborative Innovation Center for Micro/Nano Fabrication, Device and System, Tsinghua University, Beijing 100084, China; 2 State Key Laboratory of Precision Measurement Technology and Instrument, Tsinghua University, Beijing 100084, China; 3 Department of Precision Instrument, Tsinghua University, Beijing 100084, China; E-Mail: zjj.zhaojingjing@gmail.com

**Keywords:** energy harvester, wearable sensors, power supply, wearable energy harvester

## Abstract

Harvesting mechanical energy from human motion is an attractive approach for obtaining clean and sustainable electric energy to power wearable sensors, which are widely used for health monitoring, activity recognition, gait analysis and so on. This paper studies a piezoelectric energy harvester for the parasitic mechanical energy in shoes originated from human motion. The harvester is based on a specially designed sandwich structure with a thin thickness, which makes it readily compatible with a shoe. Besides, consideration is given to both high performance and excellent durability. The harvester provides an average output power of 1 mW during a walk at a frequency of roughly 1 Hz. Furthermore, a direct current (DC) power supply is built through integrating the harvester with a power management circuit. The DC power supply is tested by driving a simulated wireless transmitter, which can be activated once every 2–3 steps with an active period lasting 5 ms and a mean power of 50 mW. This work demonstrates the feasibility of applying piezoelectric energy harvesters to power wearable sensors.

## 1. Introduction

Wearable sensors are becoming smaller and increasingly widely used, resulting in an increasing need for independent and compact power supplies. Electrochemical batteries, the most common power supplies for wearable sensors, cannot meet the need because of their limited energy storage capacity and potential environmental and health risks, emerging as a critical bottleneck for wearable sensors. This has driven the development of wearable energy harvesters, which harvest the mechanical energy dissipated in human motion to provide renewable and clean energy [1]. Several concepts of wearable energy harvesters based on different mechanisms have been studied, such as electromagnetic [2–5], electrostatic [6], thermoelectric [7], nano-triboelectric [8] and piezoelectric [9–13]. Piezoelectric energy harvesters and nano-triboelectric generators can convert mechanical energy into electric energy directly, thus their structures are more compact and simpler in comparison to those of other types. The materials for nano-triboelectric generators are generally not accessible in the market, hence this work focuses on piezoelectric energy harvesters. Lead zirconate titanate (PZT) and polyvinylidene difluoride (PVDF) are the two most important piezoelectric materials for energy harvesting, owing to their high piezoelectric performance. PZT is rigid, brittle, and heavy, bringing limitations in wearable applications where flexibility is necessary. PVDF has considerable flexibility, good stability, and is easy to handle and shape [14]. Taking into account the human motion characteristics of high amplitude and low frequency, PVDF is more appropriate for wearable applications than PZT. PVDF has been used in wearable energy harvesters that are implemented in shoes [15,16], bags [17,18], and clothing [19–21]. Kymissis, *et al.* [16] developed an insole made of eight-layer stacks of 28 μm PVDF sheets with a central 2 mm flexible plastic substrate. It harnessed the parasitic energy in shoes and the average power reached 1.1 mW at 1 Hz. Granstrom, *et al.* [17] utilized PVDF straps as backpack shoulder straps to collect mechanical energy produced by the backpack, with an average power of 45.6 mW during a walking of 0.9–1.3 m/s. Yang and Yun [21] fabricated a PVDF shell structure generating an output power of 0.87 mW at a folding angle of 80° and a folding-and-unfolding frequency of 3.3 Hz, which could be worn on the elbow joint to harvest energy from human motion.

The mechanical energy dissipated in shoes can even power a computer, serving as an attractive energy source for wearable harvesters [1]. This paper develops a shoe-embedded piezoelectric energy harvester, which can be integrated in a shoe readily for energy harvesting from human locomotion with little discomfort for the wearers. The harvester is based on a specially designed sandwich structure, resulting in a thin geometrical form, a high performance and an excellent durability. Two harvester prototypes are made and tested. The first one is made up of a multilayer PVDF film and a structure of engineering plastics, which is placed under the heel. The second one is designed as an insole shape and used as a normal insole, consisting of a structure of flexible silicone rubber and two multilayer PVDF films. More power can be generated by the former prototype, while the other one has an advantage of remarkable comfort. In order to store the harvested energy and provide a constant DC output voltage, a power management circuit is designed. A series of experiments are performed to characterize the harvester prototypes, proving that the harvester can serve as a wearable power supply for low power wearable sensors and potentially provide a valuable alternative to the use of batteries.

## 2. Harvester Design

**Figure 1.**
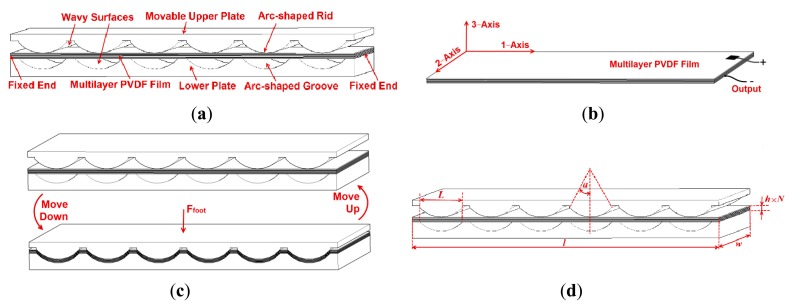
(**a**) The sandwich structure of the harvester; (**b**) The multilayer PVDF film; (**c**) The force applied by foot drive the upper plate to move up and down circularly; (**d**) The design parameters.

The main structure of the harvester is a sandwich structure, where a multilayer PVDF film is sandwiched between two wavy surfaces of a movable upper plate and a lower plate, as shown in Figure 1a. The multilayer PVDF film (Figure 1b) is fixed on the lower plate, and composed of several PVDF layers which are wired in parallel for a high output current. When the upper plate is subject to a compressive force produced by foot, the upper plate moves down and the PVDF film is stretched along 1-axis simultaneously, as presented in Figure 1c. This leads to a piezoelectric field created inside every PVDF layer, driving the free electrons in the external circuit to accumulate on the upper and lower 3-axis surfaces (electrodes) of every PVDF layer to screen the piezo-potential. When the force is lifted, the upper plate moves up and the PVDF film is relaxed, therefore the piezo-potential diminishes, resulting in releasing the accumulated electrons. A dynamic force *F_foot_* applied by foot on the upper plate drives the electrons in the external circuit to flow back and forth with an alternating current (AC) output. The sandwich structure is characterized by the inner wavy surfaces, where arc-shaped grooves and arc-shaped ribs exist. The specially designed surfaces enable the PVDF film to generate a large longitudinal deformation and reduce the harvester thickness, which enhances the harvesting performance and makes it possible to integrate the harvester into a shoe whose inner space is limited. The design parameters are defined in Table 1 and presented in Figure 1d.

**Figure 2.**
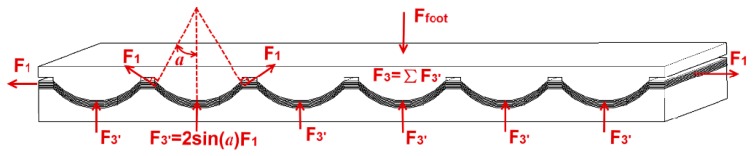
The resistive force *F_3_* against the upper plate is produced by the PVDF film.

An optimization method is utilized to design the harvester, and the objective function, constraint conditions and value ranges of parameters will be established. When the upper plate moves down to the lowest position, both the tension of the multilayer PVDF film *F_1_* and the resistive force *F_3_* against the upper plate produced by the PVDF film reach maximum. The tension *F_1_* can be expressed by:

(1) $$F_{1}=NA_{1}\sigma_{1}$$

(2) $$\sigma_{1}=\varepsilon_{1}Y$$

(3) $$\varepsilon_{1}=\left[\frac{\alpha L}{\sin\alpha }\text{INT}\left(l/L\right)-l\right]/l\approx \left[\frac{\alpha L}{\sin\alpha }\left(l/L\right)-l\right]/l=\left(\frac{\alpha }{\sin\alpha }-1\right)$$

where *σ_1_* is the normal stress, *ε_1_* is the normal strain. For simplicity of description, the frictions between the PVDF film and the two wavy surfaces are ignored, and the resistive force *F_3_* (Figure 2) can be given in Equation (4).

(4) $$F_{3}=\text{INT}\left(l/L\right)\cdot 2F_{1}\sin\alpha \approx \left(l/L\right)\cdot 2F_{1}\left(1-\cos\alpha \right)=2\frac{\text{Nwhl}}{L}\left(\alpha -\sin\alpha \right)Y$$

The quantity of charges produced by the PVDF film during the period that the upper plate moves down to the lowest position is equal to the quantity of charges generated during the period that the upper plate moves up to the initial position. According to the piezoelectric effect, the charge quantity |*Q*| is written below, and *d_31_* is the piezoelectric constant:

(5) $$\left|Q\right|=N\left(d_{31}\sigma_{1}A_{1}\frac{l}{h}\right)\approx d_{31}NA_{3}\left(\frac{\alpha }{\sin\alpha }-1\right)Y$$

|*Q*| is the key parameter to describe the energy harvesting performance of the piezoelectric harvester, and the larger |*Q*| the better. Hence, Equation (5) is the objective function for the design optimization. In Equations (3)–(5), INT(*l*/*L*) is approximated as *l*/*L*, leading to the approximate expressions of *ε_1_*, *F_3_*, and |*Q*|. At the cost of accuracy, these expressions reduce calculation cost and are deemed sufficient for the harvester design where high precision is not required. In order to improve the durability of the multilayer PVDF film, the wavy surfaces must be carefully designed to keep the PVDF film from experiencing plastic deformation. Moreover, another two factors need taking into account when designing the harvester. The first is that the resistive force *F_3_* should be lower than the driving force *F_foot_*. The second is that the harvester thickness should be less than a set value *T_m_* for a thin geometrical form of the harvester. Therefore, the constraint conditions for the design are presented in the equation below:

(6) $$\{\begin{array}{ll} \varepsilon_{1} & \approx \left(\frac{\alpha }{\sin\alpha }-1\right)\leq \varepsilon_{e}\\ F_{3} & \approx 2\frac{\text{Nwhl}}{L}\left(\alpha -\sin\alpha \right)Y\leq F_{\text{foot}}\\ T_{S} & =Nh+2\left(\frac{L}{2}\cdot \frac{1-\cos\alpha }{\sin\alpha }\right)=Nh+L\text{tan}\left(\frac{\alpha }{2}\right)\leq T_{m} \end{array}$$

where *ε_e_* is the elastic limit of the PVDF film, *T_S_* is the sum of the thicknesses of the multilayer PVDF film and a groove and a rib. The value ranges of the above design parameters can be determined by the application requirements of a specific design.

## 3. Fabrication

Two prototypes of the harvester are fabricated for two different purposes. Prototype 1 is made up of a multilayer PVDF film and a structure of rigid engineering plastics, for a high output power. Prototype 2 is designed as an insole shape, consisting of a structure of flexible silicone rubber and two multilayer PVDF films, with an advantage of excellent comfort. The multilayer PVDF film is composed of a stack of several PVDF layers connected in parallel. The properties of the PVDF layers are listed in Table 2. In order to provide a DC source for electronics, a power management circuit is utilized.

### 3.1. Fabrication of Prototype 1

**Figure 3.**
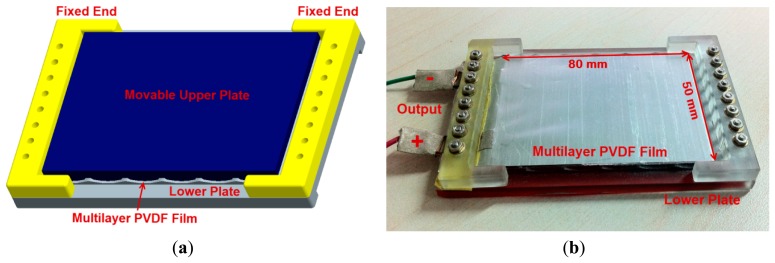
(**a**) The schematic of Prototype 1; (**b**) Prototype 1 without the movable upper plate.

Prototype 1 is designed to exploit the high pressure exerted in heel strikes. The schematic of Prototype 1 is shown in Figure 3a. The upper plate and the lower plate are made of engineering plastics whose stiffness is far greater than that of the PVDF film. The multilayer PVDF film is bolted to the lower plate, as presented in Figure 3b. According to Table 2, the elastic region of the PVDF layer is about 0%–2%. Hence, the normal strain *ε_1_* should be no more than 2%. The dynamic foot pressure distribution is studied in reference [22], showing that the peak force of a heel strike is about 400 N. Thus the resistive force *F_3_* is better no more than 400 N. The total thicknesses *T_S_* is set to not exceed 3 mm. Equation (6) indicates that *α* reaches the maximum value of 19.7° when *ε_1_* equals to 2%. The value ranges of *N*, *L* and *α* are defined in Equation (7). Besides, the PVDF film length *l* is 80 mm and the width *w* is 50 mm for a normal heel size. The harvester is designed by using optimization method. In conjunction with Matlab built-in optimization routines, several solutions are obtained. Among them, it is found that the solution of *L* = 10 mm, *N* = 8 as well as *α* = 19.7° is the optimal solution, which is selected for Prototype 1:

(7) $$\{\begin{array}{l} 1\leq N\leq 8\\ 15{}^{\circ}\leq \alpha \leq 20{}^{\circ}\\ 5\text{mm}\leq L\leq 20\text{mm} \end{array}$$

### 3.2. Fabrication of Prototype 2

**Figure 4.**
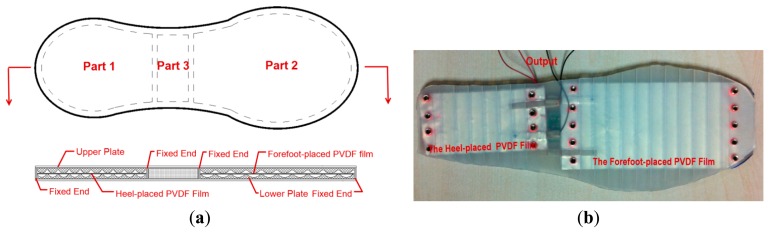
(**a**) The schematic of flexible energy harvester; (**b**) The finished Prototype 2.

Prototype 2 is designed as an insole shape, as illustrated in Figure 4a, which can be divided into three parts. Part 1 and Part 2 are the sandwich structures for harvesting energy with two 8-layer PVDF films. Similar to the rigid energy harvester, Part 1 is applied to harness the energy from heel strikes. Part 2 is used to tap the energy dissipated in bending of the shoe. Part 3 is under foot arch where foot pressure is low, which is a chamber offering vacant space to accommodate circuits and energy storage devices. The upper plate and the lower plate are made of silicone rubber (by injecting modeling process) whose stiffness is far less than that of the engineering plastics used in Prototype 1. Therefore, the practical PVDF film deformation is lower than the theoretical maximum value calculated by Equation (3). In order to reduce this adverse influence caused by the flexible material, the angle *α* is increased to 27.7°, resulting in a theoretical maximum normal strain *ε_1_* of 4%. The chord length *L* is assigned 10 mm. The upper plate, the lower plate and two multilayer PVDF films are fixed together by bolts, and the finished Prototype 2 is shown in Figure 4b.

### 3.3. Power Circuit

**Figure 5.**
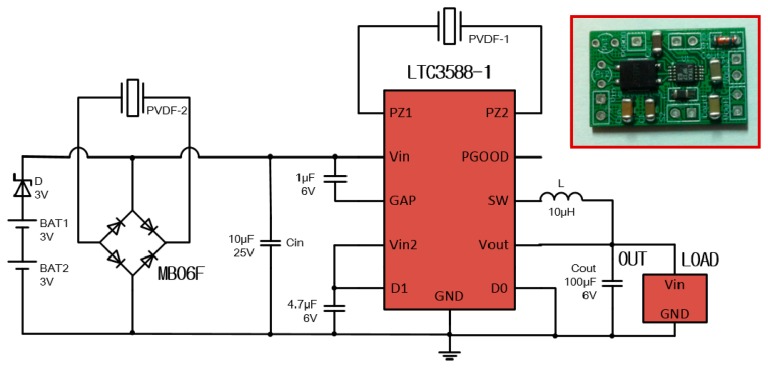
Schematic and physical photograph of the power management circuit.

The PVDF film generates AC power with high voltage and low current, which cannot meet the need of commercial electronics that are practically driven by a uniform DC power. Furthermore, if wearable sensors are not in operation, the harvested energy needs storing in a storage device. Based on the above the two considerations, a power management circuit is designed, as diagrammed in Figure 5, mainly consisting of two full bridge rectifiers, a buck converter, two button batteries (optional) and some capacitors. The buck converter and one rectifier are contained in the chip LTC 3588-1. Two rectifiers can simultaneously rectify two AC currents of different phases respectively, which is useful for Prototype 2. The harvested energy accumulates on the input capacitor *C_in_*, and then transferred by the buck converter to the output capacitor *C_out_*. The target value of the output voltage *V_out_* (the voltage across output capacitor *C_out_*) is set to 3.6 V. When *V_out_* reaches the target value, a logic high is produced on the PGOOG pin. If the load shuts off or the input power is more than the output power, there exists unused energy, which will be stored in the batteries. On the contrary, if the harvester cannot generate enough power to meet the load demand, the batteries will be discharged to offer supplemental power.

## 4. Experiments and Results

**Figure 6.**
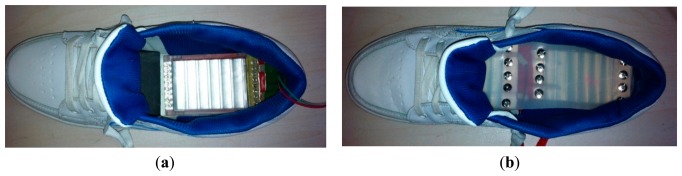
(**a**) Prototype 1 with an 8-layer PVDF film was mounted on the inner sole and gathered energy under the heel; (**b**) Prototype 2 with two 8-layer PVDF films was used as a normal insole in a shoe.

Prototype 1 and Prototype 2 were implemented in shoes to harness the parasitic energy during walking, as shown in Figure 6. A series of experiments were carried out to evaluate the performance of the prototypes. In the first experiment, the prototype was terminated with a matched resistor(s), hence yielded maximum power transfer. In the second experiment, the quantities of the charge produced by the prototypes during one step (*Q_S_*) were measured. In the third and fourth experiment, the prototypes were connected with the power management circuit (without using batteries) to form DC power supply systems. The start-up time of the systems were measured. Besides, the power supply systems were tested to power a simulated transmitter load.

### 4.1. Performance of Prototype 1

**Figure 7.**
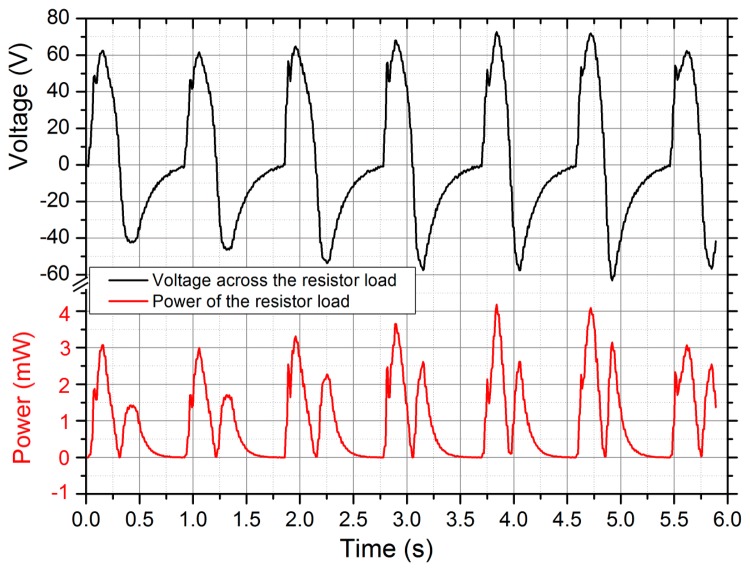
The voltage across the resistor load and the resulting power delivered to the resistor.

Prototype 1 with an 8-layer PVDF film (the capacitance of the PVDF film was 126 nF) was mounted on the inner sole and harnessed energy under the heel. Firstly, Prototype 1 was tested during a brisk walk at roughly 1 Hz [23], and terminated with a 1.268 MΩ resistor. Figure 7 shows the voltages across the resistor and the resulting power delivered to the resistor. The peak-to-peak values of the voltage were about 136 V. The peak power was roughly 4 mW, and the mean power was much lower with a value of about 1 mW, meaning that the average net energy transfer was about 1 mJ per step. It demonstrates that the harvester is an attractive alternative for powering some low wearable sensors, such as activity trackers, blood pressure sensors, and sensors for healthcare [24].

**Figure 8.**
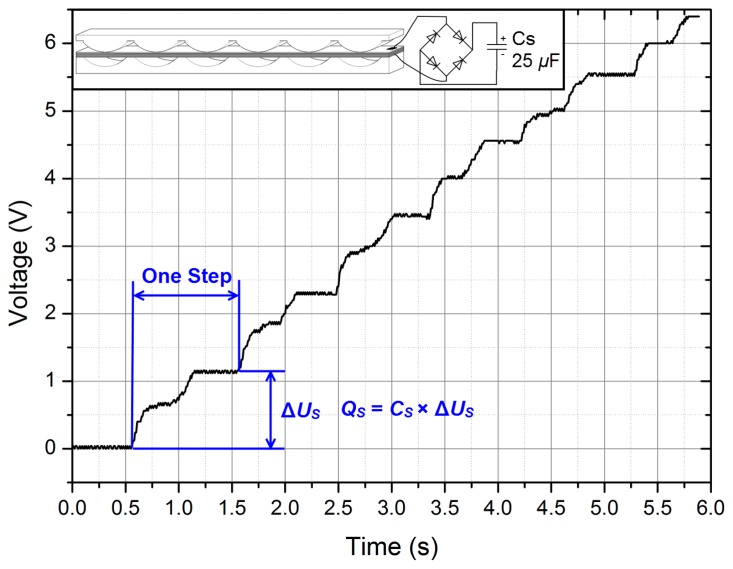
The voltage across the storage capacitor *C_S_*.

Secondly, *Q_S_* was measured by using an energy storage circuit shown in Figure 8. During one step [25], the increment of the voltage on the storage capacitor *C_S_* was Δ*U_S_*. About 30 μC charge was produced per step. If two button batteries (GMB301009 for example) with a capacity of 2 × 8 mAh are charged by Prototype 1, as shown in Figure 5, they can completely charge from empty in 1.92 million steps (about 200 days with 10,000 steps per day, 2 × 8 mA × 3600 s ÷ 30 μC/step = 1,920,000 steps ≈ 10,000 step/day × 200 day). Although it is not an optimal way to charge batteries, the battery lifetime can be prolonged owing to the harvester.

**Figure 9.**
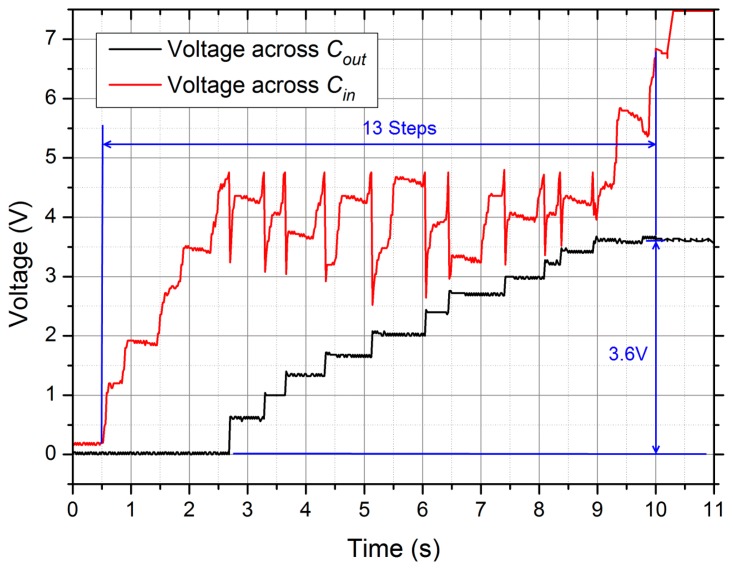
The two measured voltages across *C_in_* and *C_out_*, and after 13 steps the output voltage reached the set value 3.6 V.

Thirdly, the start-up time of DC power supply system, consisting of Prototype 1 and the power management circuit, was measured. Figure 9 shows two measured voltages on *C_in_* and *C_out_*. The voltage on *C_in_* increased during every step, and when it surpassed the threshold of about 4.8 V, the energy would be transferred to *C_out_*. Meanwhile, *C_in_* discharged and its voltage declined rapidly with an increment of the voltage on *C_out_*. It indicates that after 13 steps the voltages across *C_out_* (the output voltage *V_out_*) reached the set value 3.6 V. Hence, the setup time of the system was about 13 s during a walk of 1 Hz, which was not a long waiting time for wearers.

**Figure 10.**
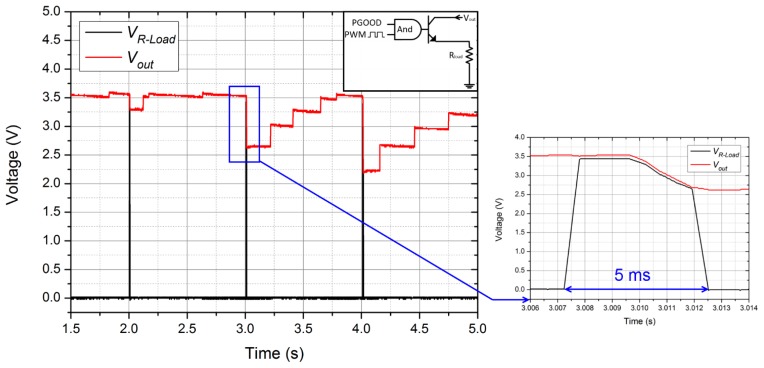
Performance of the power supply system.

Fourthly, the complete DC power supply system was tested by powering a simulated wireless transmitter [26], as shown in Figure 10. The simulated load was modeled as a 180 Ω resistor load *R_load_* which was controllable by pulse-width modulated (PWM) signal with a duty cycle of 0.5% and a frequency of 1 Hz. When the output voltage of the power supply system was ready (PGOOD at logic high), the supply rails to the simulated load was activated and energy was consumed. Prototype 1 was driven at roughly 2 Hz. Figure 10 shows the representative signals from the complete power supply system when powering the simulated load. The red trace is the output voltage *V_out_* and the black trace is the voltage across the resistor load (*V_R-load_*). The simulated transmitter load was activated once every 2–3 steps. Because the load dissipated power faster than the harvester generated power, *V_out_* and *V_R-load_* fell when the simulated load was active. During the active period of 5 ms, *V_R-load_* declined from 3.4 V to 2.7 V, and the calculated values of the mean load power and the mean load current were 50 mW and 16 mA respectively. The power output could certainly be used to power some low-power wireless transmitters, such as transmitters based on Bluetooth Low Energy (BLE) or ANT+.

### 4.2. Performance of Prototype 2

The insole-shaped Prototype 2 with two 8-layer PVDF films was placed in a shoe, and tested during a walk at about 1 Hz. The capacitances of the PVDF film placed under forefoot and the PVDF film placed under heel were 250 nF and 110 nF respectively. The four experiments executed above were also done for Prototype 2.

**Figure 11.**
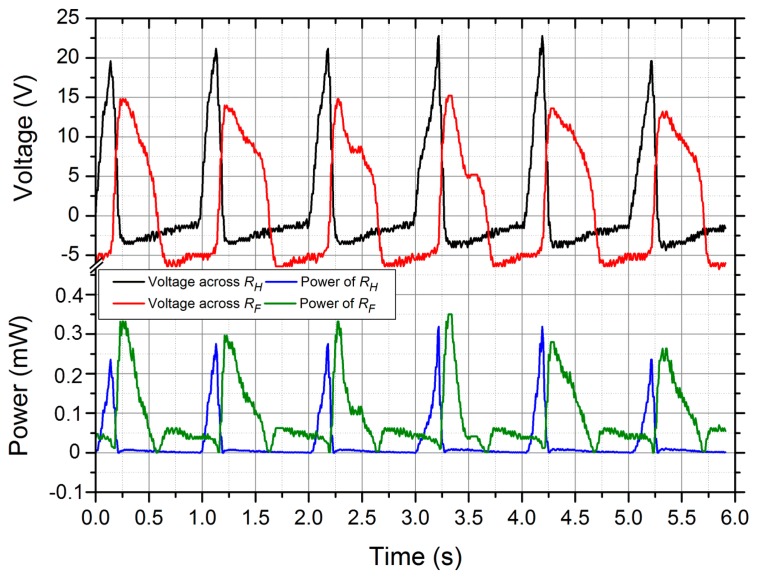
The voltages across *R_H_* and *R_F_* as well as the resulting power delivered to the resistors.

**Figure 12.**
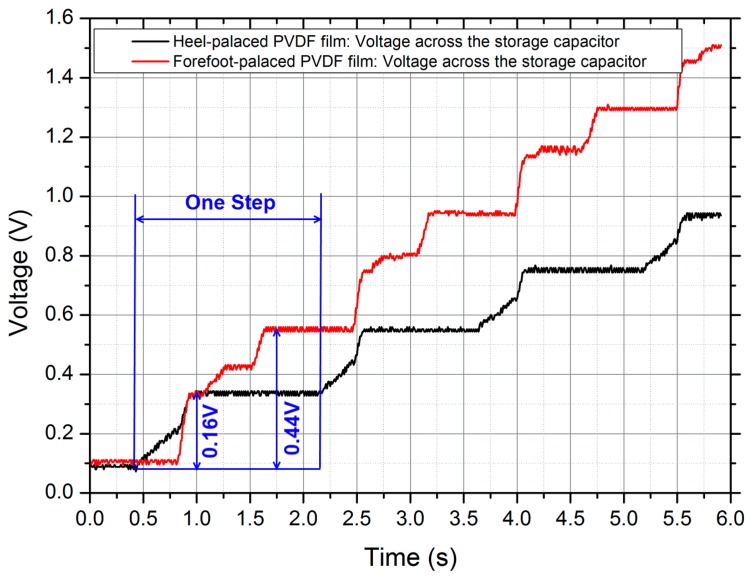
The voltages across the storage capacitors of 25 μF, the red trace for the forefoot-placed PVDF film, the black trace for the heel-placed PVDF film.

In the first experiment, the forefoot-placed PVDF film was connected with a 660 kΩ resistor *R_F_*, and the heel-placed PVDF film was terminated with a 1.682 MΩ resistor *R_H_*. The voltages across the resistors and the powers were presented in Figure 11. Obviously, the two output voltages had different phases. The heel-placed PVDF film produced peak-to-peak voltages of roughly 30 V, while the forefoot-placed PVDF film gave lower response with peak-to-peak values approaching 22 V. The mean powers were 30 μW for the heel-placed PVDF film and 90 μW for the forefoot-placed PVDF film. In total, about 0.12 mJ per step could be supplied, which was significantly lower than the energy of 1 mJ per step provided by Prototype 1. That was because the PVDF film deformations in Prototype 2 was smaller than those in Prototype 1. In the second experiment, about 5 μC charges could be generated by the heel-placed PVDF film during one step, and 11 μC for the forefoot-placed PVDF film, as shown in Figure 12. In the third experiment, the two PVDF films were wired with the two rectifiers of the power management circuit respectively, and the harvester served as a DC power supply system. The output voltage reached the set value 3.6 V after 25 steps. In the fourth experiment, the power supply system could activate the simulated load once every 6–8 steps. In conclusion, Prototype 2 offers more comfort for users compared to Prototype 1, at a cost of generating performance.

## 5. Discussion

Compared with other reported shoe-embedded PVDF energy harvesters, the harvester proposed here has advantages of a thin geometrical form, high performance and an excellent durability, benefitting from its specially designed sandwich structure. The deformation of the multilayer PVDF film is kept elastic and the maximum deformation is close to PVDF elastic limit, thus there is a tradeoff between performance and durability. The average power of the harvester is up to 1 mW (at 1 Hz), which approximates the power of 1.1 mW (at 1 Hz) of the PVDF insole in reference [15]. While more comfort and durability can be provided by our design. By combining the merits of Prototype 1 and Prototype 2, a better harvester can be developed in the future work. The arc-shaped grooves and ribs on the wavy surfaces will be made of some harder material, polyurethane for example, to improve the PVDF film deformation for more energy produced, and the other parts of the plates are made of flexible material to keep users comfortable. In addition, increasing the number of PVDF layers serves as another approach to improving generating performance.

## 6. Conclusions

A shoe-embedded piezoelectric energy harvester is developed in this paper, and it can be integrated in a shoe readily for energy harvesting from human locomotion. Two prototypes with different characteristics are fabricated and tested. One prototype produces more energy, while the other one is more comfortable without creating any inconvenience or discomfort for wearers. The DC power supply system, including the harvester and a power management circuit, is used to collect the mechanical energy dissipated in shoes and power some low-power wearable sensors, such as activity trackers. Even though the harvester is unlikely to replace completely the batteries in all wearable sensors, it is a significant role in reducing the problems related to the use of batteries. The work presents a successful attempt in harnessing the parasitic energy expended during person's everyday actions to produce power for wearable sensors.

## Figures and Tables

**Table 1.** Design parameters.

**Parameter Name**	**Descriptions**
*l*	PVDF layer length (along 1-axis)
*w*	PVDF layer width (along 2-axis)
*h*	PVDF layer thickness
*A_1_* (= *wh*)	Cross-sectional area of one PVDF layer
*A_3_* (= *wl*)	3-axis surface area of one PVDF layer
*N*	The number of PVDF layers
*L*	Chord length of an arc-shaped groove/ rib
2α	Intersection angle of an arc-shaped groove/rib
*n* (=INT(*l*/*L*))	Number of arc-shaped grooves/ribs

**Table 2.** Properties of the PVDF layer.

**Material Property**	**Symbol**	**Value**
Relative permittivity	*ε_r_*	9.5 ± 1
Piezoelectric constant	*d_31_*	17 × 10^−12^ C/N
Elastic modulus	*Y*	2500 MPa
PVDF layer thickness	*h*	30 *μ*m
Yield strength	*σ_s_*	45∼55 MPa
